# Improving Tuberculosis Vaccine Trial Efficiency: A Tough Nut to Crack

**DOI:** 10.1093/infdis/jiad360

**Published:** 2023-08-21

**Authors:** Helen McShane

**Affiliations:** The Jenner Institute, University of Oxford, Oxford OX3 7DQ, United Kingdom

**(See the Major Article by Hill et al on pages 1150–3.)**

The only licensed vaccine against tuberculosis, bacille Calmette-Guérin (BCG), was developed in 1921. More than 100 years later, the need for a more effective vaccine is more pressing than ever. Rates of drug-resistant tuberculosis are rising and the coronavirus disease 2019 (COVID-19) pandemic had a devastating effect on tuberculosis control programs, reversing improvements in mortality and morbidity seen in recent years [1].

Tuberculosis vaccine development is extremely challenging, in part because *Mycobacterium tuberculosis* is a highly complex organism, adept at evading and subverting the host protective immune response. We do not fully understand what constitutes a protective immune response. Validated immune correlates of protection with which to guide vaccine research and development are lacking. Modeling the real-world environment of exposure to common coinfections such as human immunodeficiency virus (HIV), helminths, nontuberculous mycobacteria and cytomegalovirus, which may influence vaccine efficacy, is not feasible in preclinical animal models and the predictive value of animal models for human efficacy is unclear.

There is currently no substitute for human efficacy testing—but efficacy trials with new tuberculosis vaccine candidates are costly and take years to enroll, even in the highest-burden settings. We need to explore more efficient ways to conduct these trials and demonstrate human efficacy. Using *M. tuberculosis* infection, rather than tuberculosis disease, as an end point, may be one way to achieve this [2]. However, whilst infection is a prerequisite for disease, the relationship between immunity against infection and immunity against disease is unclear. Using a prevention of infection efficacy end point as a stage gate for a prevention of disease trial may lead to the premature rejection of an effective tuberculosis vaccine.

Using lessons learned from the COVID-19 pandemic, Hill et al offer in a Perspective several suggestions for how a tuberculosis vaccine efficacy trial could be conducted more efficiently [3]. They focus on 3 elements of trial design: study size, participant characteristics, and enrolment procedures, and bring some of these elements together in an alternative trial design, focused on increased sample size and reduced enrolment time.

Cost and logistic constraints mean that a significantly increased sample size could only feasibly be achieved with a simpler, less intensive trial design, such as the proposal to utilize a more passive case finding approach using strengthened local health care systems, rather than the more active follow-up strategies usually used in trials with unlicensed and investigational products. Such an approach would have the added advantage of improving tuberculosis case detection more generally in the communities within which phase 3 trials are conducted. However, active follow-up in a phase 3 trial designed to support a regulatory filing for registration is necessary for 2 reasons. First, it is critical to ensure there is a sufficient safety database for licensure, and that rare but serious adverse events are detected during the trial. Such events are likely to be less detectable using a more passive follow-up. Second, stringency of primary end points is necessary in a phase 3 trial to ensure confidence in the specificity of the end points, and that every end point is captured. Both diagnostic stringency and complete case ascertainment are both less likely in a passive follow-up setting where the cases will be defined and diagnosed in the routine health care setting. Whilst such a passive approach to follow-up was utilized in a large 200 000-subject BCG revaccination trial in Brazil, this trial was evaluating a licensed vaccine, delivered by the licensed route, with an enormous preexisting safety database [4]. Such an approach with an unlicensed vaccine without such a safety database is less appropriate as infrequent serious adverse events may be missed. Conducting enhanced follow-up in only a proportion of subjects, as suggested by the authors, would be unlikely to be sufficient for regulatory approval and licensure. In the BioNTech COVID-19 vaccine trial, the safety monitoring was in line with what would be conventional for licensure of a novel vaccine candidate, with the Food and Drug Administration-defined main safety subset being 37 706 (87%) of the total 43 448 participants enrolled [5]. An end of trial prevalence survey in enrolled participants would add significant cost and complexity, and would result in many cases of tuberculosis only being detected very late. Whilst such an approach was acceptable in the Chingleput trial in 1968, where there was already a significant safety database with BCG, such an approach is unlikely to be acceptable in today's ethical and regulatory environment.

The cost and complexity of strengthening local tuberculosis control programs, working with local governments, etc., would, even if practical across the diverse political and health care landscapes involved, reduce any financial advantage over the more conventional focused follow-up strategy.

Key to this issue is the requirement for any phase 3 trial design to be compliant with regulatory requirements for licensure. The BCG revaccination trial was able to utilize a simple trial design and passive follow-up because the intervention being evaluated was an already licensed product. Likewise, the RECOVERY trial platform, which evaluated the efficacy of already licensed drugs against COVID-19, utilized a simple trial design to facilitate participation across multiple sites, ensuring large numbers of subjects could be enrolled [6]. It is highly unlikely that either of these suggested approaches could be used to support a licensure trial where an investigational product was being evaluated, such as in an efficacy trial with a novel tuberculosis vaccine candidate.

The authors acknowledge that the scientific disadvantages of enrolling participants with increased risk of developing tuberculosis, either through recent exposure or because of comorbidities such as HIV infection and diabetes mellitus, outweigh any potential benefits. Recently exposed people and those with risk factors are often eligible for chemoprophylaxis, which mitigates the increased risk of infection and could not be reasonably withheld in a clinical trial. Selective enrolment of people with comorbidities may mask vaccine efficacy—a vaccine may not protect, or may protect less well, in these high-risk groups. Furthermore, whilst the risks of tuberculosis in those with HIV coinfection was significantly increased in a preantiretroviral era, this increased risk is much smaller in the context of well-controlled HIV infection. Modeling studies to quantify the impact of this suggestion would likely demonstrate that the limitations outweighed the theoretical, face value benefit.

The “large simple” design with more passive-style case finding would also preclude the collection of immune correlate samples. Given the first new licensed tuberculosis vaccine is unlikely to demonstrate the near 100% protection seen with the COVID-19 and human papillomavirus vaccines, we will need to continue to develop improved tuberculosis vaccines. A validated immune correlate of protection would be game changing in tuberculosis vaccine development and we should continue to maximize the possibility of deriving this from every efficacy trial.

Some of the approaches suggested in this Perspective to reduce enrolment time are worthy of consideration. Replacing sputum culture at screening with the newer molecular methods could expedite enrolment and, as Hill et al suggest [3], enrolling regardless of interferon γ release assay (IGRA) status would improve the generalizability of any findings. However, the issue with enrolling without at least stratifying by IGRA status and limiting the proportion of IGRA-negative participants is that risk of tuberculosis in IGRA-negative participants is extremely low. It would not be feasible to conduct an efficacy trial with the numbers required if the majority of participants were IGRA negative.

The COVID-19 pandemic inspired us with what can be possible when funders and scientists around the world unite against a new pathogen. *M. tuberculosis* is a wily adversary with centuries of experience to outfox us. No breakthroughs are likely without determined, sustained, investment in research and development and a concerted global effort to design and conduct efficacy trials, such as that seen with the recent announcement of a funder collaboration to mobilize the large-scale funding required for the M72 phase 3 trial [7]. It is only with this concerted global effort that we stand a chance of achieving the sustainable development goal of eliminating tuberculosis as a global public health problem—a goal that will only be possible with a highly effective tuberculosis vaccine.

## Notes

***Financial support*.** HMcSis a Wellcome Trust Investigator (WT 206331/Z/17/Z) and receives support from the National Institute for Health and Care Research Oxford Biomedical Research Centre.

***Potential conflict of interests*.** The author: No reported conflicts of interest. The author has submitted the ICMJE Form for Disclosure of Potential Conflicts of Interest.
